# Behavioral roles of biogenic amines in bumble bee males

**DOI:** 10.1038/s41598-022-25656-7

**Published:** 2022-12-05

**Authors:** Tomohiro Watanabe, Ken Sasaki

**Affiliations:** Graduate School of Agriculture, Tamagawa University, Machida, Tokyo, 194-8610 Japan

**Keywords:** Social evolution, Neurophysiology, Animal behaviour, Animal physiology

## Abstract

To compare the behavioral roles of biogenic amines in the males of primitive and advanced eusocial bees, we determined the levels of dopamine- and octopamine-related substances in the brain, and the behavioral effects of these monoamines by drug injection in the primitive eusocial bumble bee, *Bombus ignitus*. The levels of dopamine and its precursors in the brain peaked at the late pupal stage, but the dopamine peak extended to adult emergence. The tyramine and octopamine levels increased from the mid-pupal to adult stages. The locomotor and flight activities, and light preference increased with age. Injection of octopamine and its receptor antagonist had significant effects on the locomotor and flight activities, whereas dopamine injection did not, indicating that these activities can be regulated by the octopaminergic system. We also determined the dynamics of dopamine-related substances in honey bee (*Apis mellifera*) drones. The changes in the dopamine level in the brains of honey bee drones exhibited two peaks from the pupal to adult stages, whereas the bumble bee males had only one peak. These are consistent with the behavioral functions of dopamine in honey bee drones and ineffectiveness of dopamine injection at the adult stage in bumble bee males.

## Introduction

Eusociality is the most complex social system, with division of labor among genetically related individuals, and it has been reported in various species in a wide range of insect taxa^1–4^. In eusocial hymenopterans, social behaviors involving cooperation among colony members are only observed in females. Male eusocial hymenopterans specialize in reproduction and they do not participate in nest construction, brood care, or colony defense^1,2,5^, although males in eusocial bees contribute to colonial thermoregulation^6,7^. Therefore, the behavioral repertories of males are much smaller than those of workers. Investigating the behaviors of males and the associated physiological mechanisms may provide important insights into the specialization of behavior for reproduction in males in highly social environments.

In advanced eusocial environments, the behaviors of honey bee (*Apis mellifera*) drones have been investigated in various research fields, including genetics, endocrinology, reproductive biology, and evolutionary biology^8^. Honey bee drones mature sexually for a week after emergence and start flying to mate with virgin queens^5,9^. The locomotor and flight activities associated with reproduction in drones increase with age during their sexual maturation^10^. Honey bee drones return to the mother colony, whereas bumble bee males do not learn the colony’s location and never return to the colony^7,11,12^. After leaving the nest, bumble bee males forage for themselves while searching for gynes to mate. This may be due to the male production at the end of the colony season when the nutritional status of the bumble bee colonies declines. Thus, the dependence of males on the colony differs between the honey bee and bumble bee, which might reflect the adaptation of male behavior to different social environments.

Biogenic amines are physiological agents that function as neurotransmitters, neuromodulators, and neurohormones in various insect species^13–17^. These substances are involved in the age-related division of labor in honey bee workers^18,19^ and the reproductive behavior of virgin queens^20,21^. In honey bee drones, the levels of dopamine and octopamine in the brain increase with age^10,22,23^. Injecting these monoamines into the hemolymph of drones enhances their locomotor and flight activities^10,23^. Therefore, the increases in the levels of dopamine and octopamine in the brain may be closely related to mating flight activities in honey bee drones. Dopamine also enhances the locomotor and flight activities in males of the solitary bee *Xylocopa appendiculata*^24^, which shares a common ancestor in Apidae^25^. Thus, the regulation of male reproductive behavior by biogenic amines might be shared among eusocial bees and should be tested in other species within a monophyletic clade, such as the corbiculate bees.

Bumble bees comprise a large group of primitive eusocial bees, and they are ideal species for investigating the missing link between solitary and advanced eusocial species, although the multidimensional analysis of social behaviors suggests the small difference of social complexity between honey bees and bumble bees^26^. Male bumble bees are produced during the late summer to early autumn season by fully grown colonies in temperate regions^2,7^. The major role of bumble bee males in the colony is reproduction, and they can mate with different gynes in contrast to single mating honey bee drones. Bumble bee males complete their sexual maturation 6–9 days after emergence and initiate mating with gynes^27^. Elucidating the physiological mechanisms that underlie the reproductive behavior of bumble bee males and comparing them with those in the honey bee may improve our understanding of the adaptation of regulation system of male reproductive behavior to social environment. However, no previous studies have reported the regulation of reproductive behavior by biogenic amines in bumble bee males. Thus, in the present study, we investigated the dynamics of the levels of biogenic amines in the brains and effects of biogenic amines on reproductive behavior in bumble bee (*B. ignitus*) males. We also compared the mechanisms in the bumble bee and honey bee to determine whether the mechanisms mediated by biogenic amines differ between the two species.

## Results

### Changes in the levels of biogenic amines in the brains of bumble bee males

**Figure 1:**
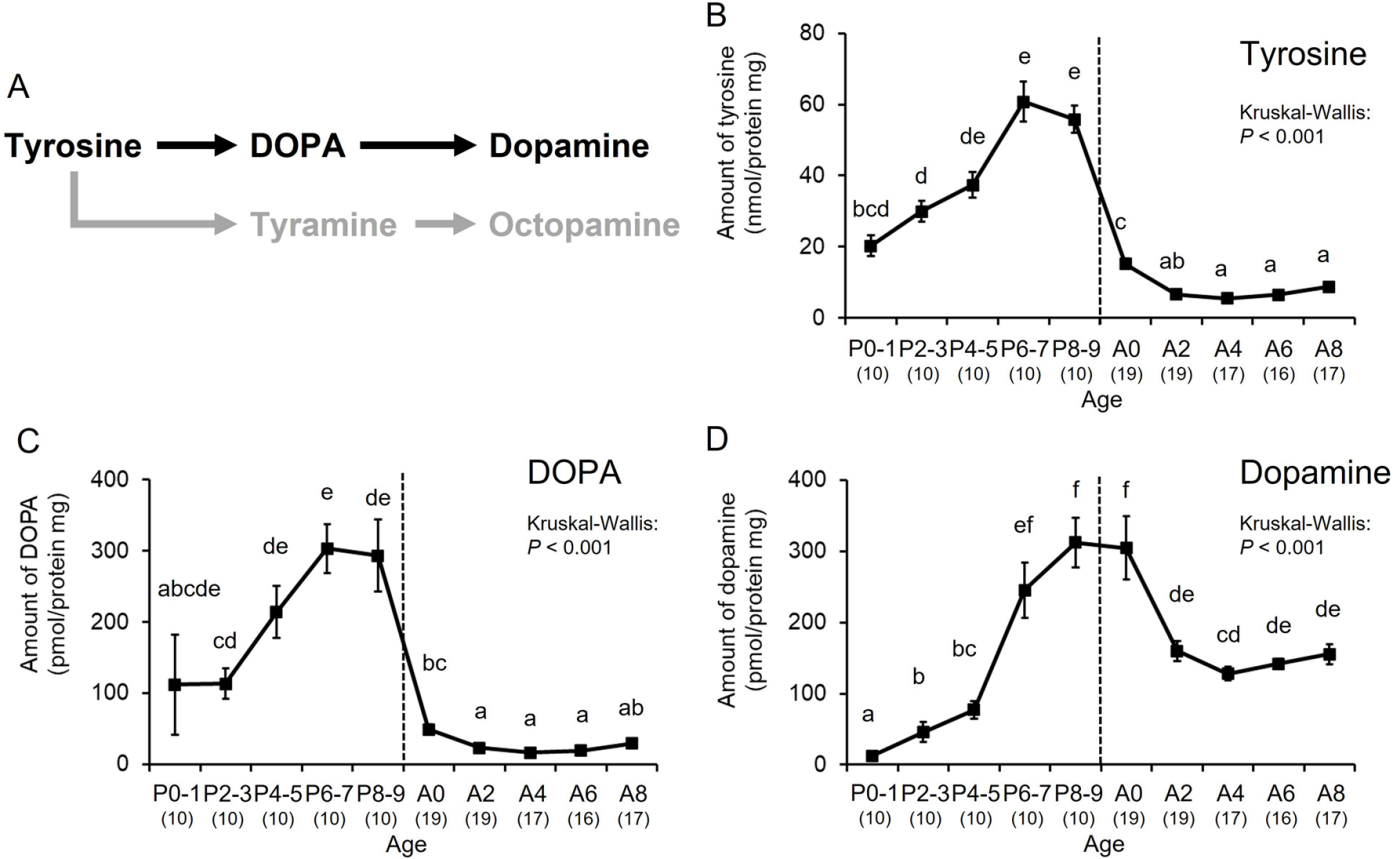
Age-dependent changes in dopamine-related substances normalized by brain protein in bumble bee males. (**A**) Synthetic pathway for dopamine-related substances. Levels are shown of tyrosine (**B**), DOPA (**C**), and dopamine (**D**) in the brain. Error bars indicate standard errors. Significant differences according to the Steel–Dwass test (*P* < 0.05) are indicated by different letters. Sample sizes are shown in parentheses.

We initially determined the levels of dopamine, octopamine and their precursors in the brains of bumble bee male pupae and adults by using high-performance liquid chromatography with electrochemical detection (HPLC-ECD) systems. The levels of two dopamine precursors (tyrosine and DOPA, Fig. 1A) increased with age during the pupal stage (P0–1 to P6–7), but decreased before emergence (P8–9 to A0) and then remained at constant lower levels during the adult stage (A2 to A8) compared with those during the pupal stage (tyrosine: Kruskal–Wallis: *H* = 106.500, *df* = 9, *P* < 0.001, Steel–Dwass: *P* < 0.05, Fig. 1B; DOPA: Kruskal–Wallis: *H* = 88.300, *df* = 9, *P* < 0.001, Steel–Dwass: *P* < 0.05, Fig. 1C). However, the dopamine levels increased with age during the pupal stage, and remained at the highest levels from the end of the pupal state (P8–9) to adult emergence (A0), then decreasing (A2–A8) (Kruskal–Wallis: *H* = 88.599, *df* = 9, *P* < 0.001, Steel–Dwass: *P* < 0.05, Fig. 1D). The changes in the dopamine levels were basically similar to those in the tyrosine and DOPA levels, but the decrease in the dopamine level after emergence was delayed compared with the decreases in the dopamine precursors.

**Figure 2:**
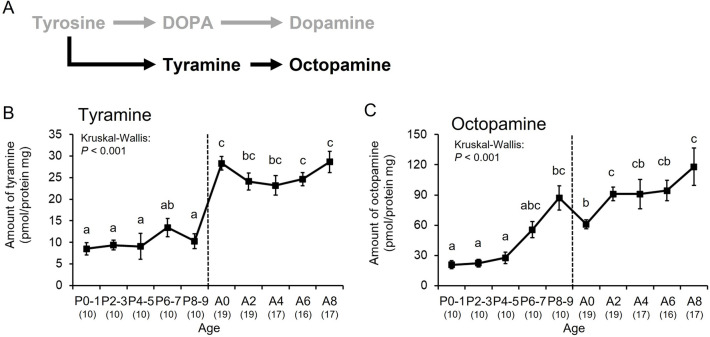
Age-dependent changes in octopamine-related substances normalized by brain protein in bumble bee males. (**A**) Synthetic pathway for octopamine-related substances. Levels are shown of tyramine (**B**) and octopamine (**C**). Error bars indicate standard errors. Significant differences according to the Steel–Dwass test (*P* < 0.05) are indicated by different letters. Sample sizes are shown in parentheses.

Tyramine is a metabolite of tyrosine, a precursor of octopamine and has its own function in insects^15,28^ (Fig. 2A). The levels of tyramine remained constant during the pupal stage, increased from the end of the pupal stage (P8–9) to adult emergence (A0), and remained at higher levels during the adult stage (A0–A8) than the pupal stage (Kruskal–Wallis: *H* = 79.481, *df* = 9, *P* < 0.001, Steel–Dwass: *P* < 0.05, Fig. 2B). The octopamine levels increased from the mid-pupal stage (P4–5) to the adult stage (Kruskal–Wallis: *H* = 80.097, *df* = 9, *P* < 0.001, Steel–Dwass: *P* < 0.05, Fig. 2C). Thus, the levels of two phenolamines (tyramine and octopamine) derived from tyrosine increased gradually with age from the mid-pupal to adult stages.

### Age-related changes in behavioral activity in bumble bee males

**Figure 3:**
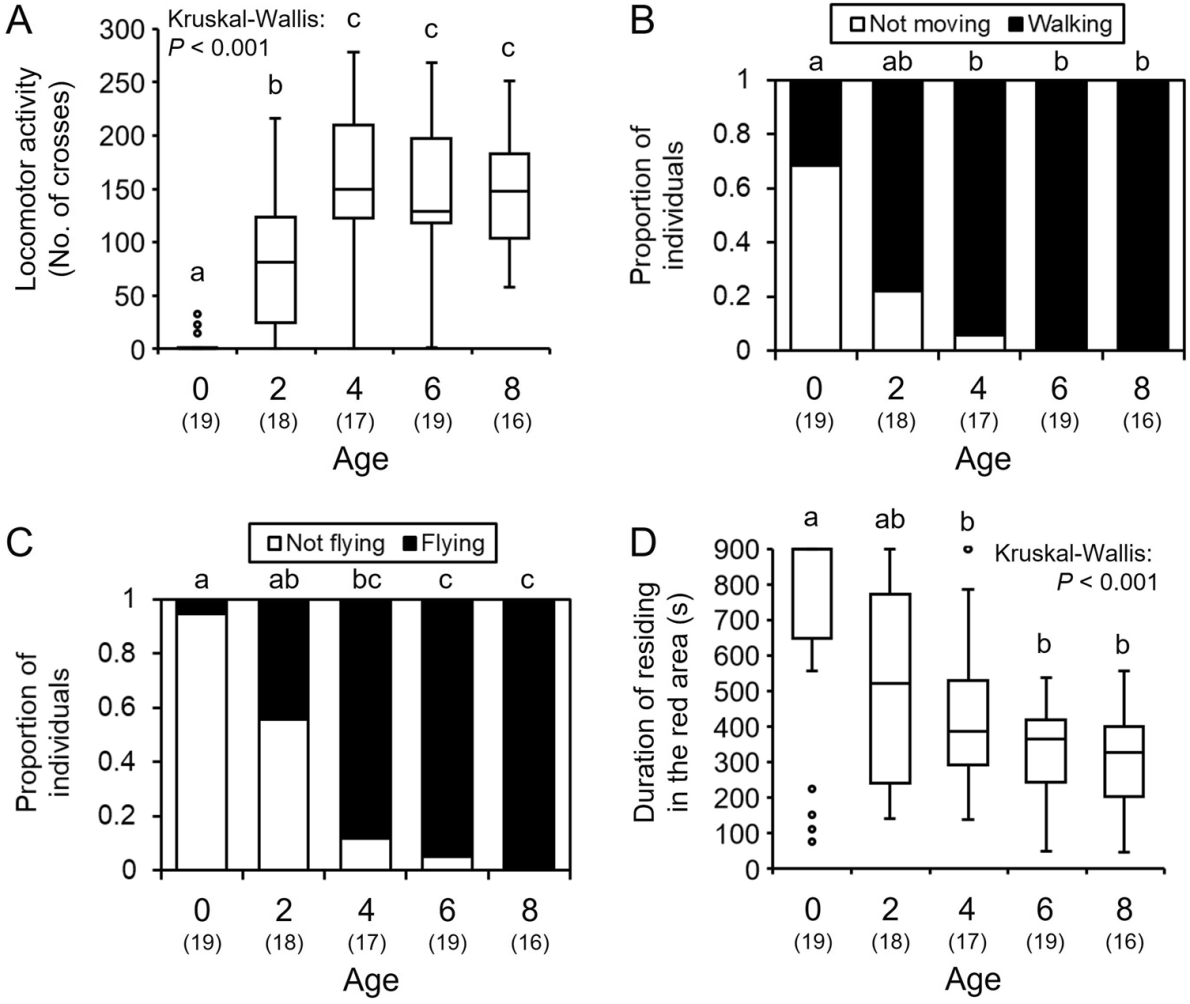
Age-dependent changes in behavioral activities in bumble bee males: (**A**–**B**) spontaneous locomotor activity, (**C**) spontaneous flight activity, and (**D**) light preference indicated by duration of residing in the red area. Locomotor activity and duration of residing in the red area are indicated by box plots. Significant differences according to the Steel–Dwass test (*P* < 0.05) or Fisher’s exact test with Bonferroni correlation (P < 0.05) are indicated by different letters. Sample sizes are shown in parentheses.

To investigate the changes in behavioral activities with age, the locomotor and flight activities of bumble bee males were measured at different ages. The locomotor activity increased significantly from A0 to A4 and then remained at constant higher levels (Kruskal–Wallis: *H* = 46.139, *df* = 4, *P* < 0.001, Steel–Dwass: *P* < 0.05, Fig. 3A). The proportion of individuals that walked in the ring-shaped chamber increased from A0 to A4, with up to 100% at A6 and A8 (Fisher’s exact test with Bonferroni correction: *P* < 0.05, Fig. 3B). The flight activity increased significantly with age in parallel with the locomotor activity (Fisher’s exact test with Bonferroni correction: *P* < 0.05, Fig. 3C). The duration of residence in the red area decreased significantly with age (Kruskal–Wallis: *H* = 19.634, *df* = 4, *P* < 0.001, Steel–Dwass: *P* < 0.05, Fig. 3D), thereby indicating that males gradually preferred the light area with age.

### Effects of dopamine on behavioral activity in bumble bee males

**Figure 4:**
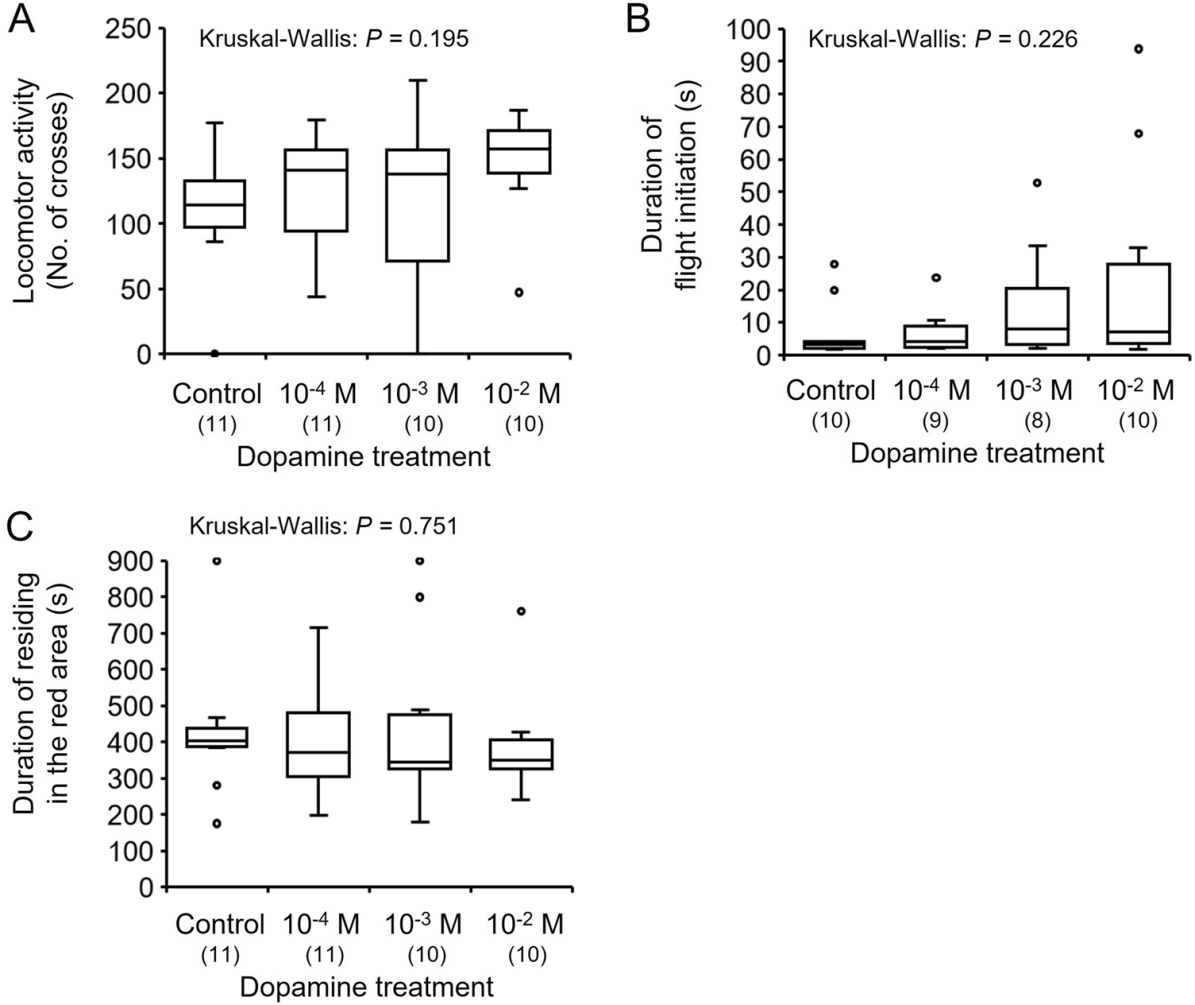
Effects of dopamine injection on behavioral activities of 4-day-old males in the bumble bee: (**A**) spontaneous locomotor activity, (**B**) duration of flight initiation, and (**C**) duration of time residing in the red area as a light preference index. Locomotor activity, duration of initiation, and duration of residing in the red area are indicated by box plots. Sample sizes are shown in parentheses.

To determine effects of dopamine on behavior, we measured the locomotor and flight activities and light preference on dopamine injected males. The locomotor activity did not differ significantly between the control and dopamine-injected individuals (Kruskal–Wallis: *H* = 4.705, *df* = 3, *P* = 0.195, Fig. 4A). The latency of flight initiation did not differ significantly between the control and dopamine-injected individuals (Kruskal–Wallis: *H* = 4.354, *df* = 3, *P* = 0.226, Fig. 4B). The duration of residence in the red area did not differ significantly between the control and dopamine-injected individuals (Kruskal–Wallis: *H* = 1.209, *df* = 3, *P* = 0.751, Fig. 4C). Thus, dopamine injection did not affect the locomotor and flight activities and response to light in males.

### Effects of octopamine on behavioral activity of bumble bee males

**Figure 5:**
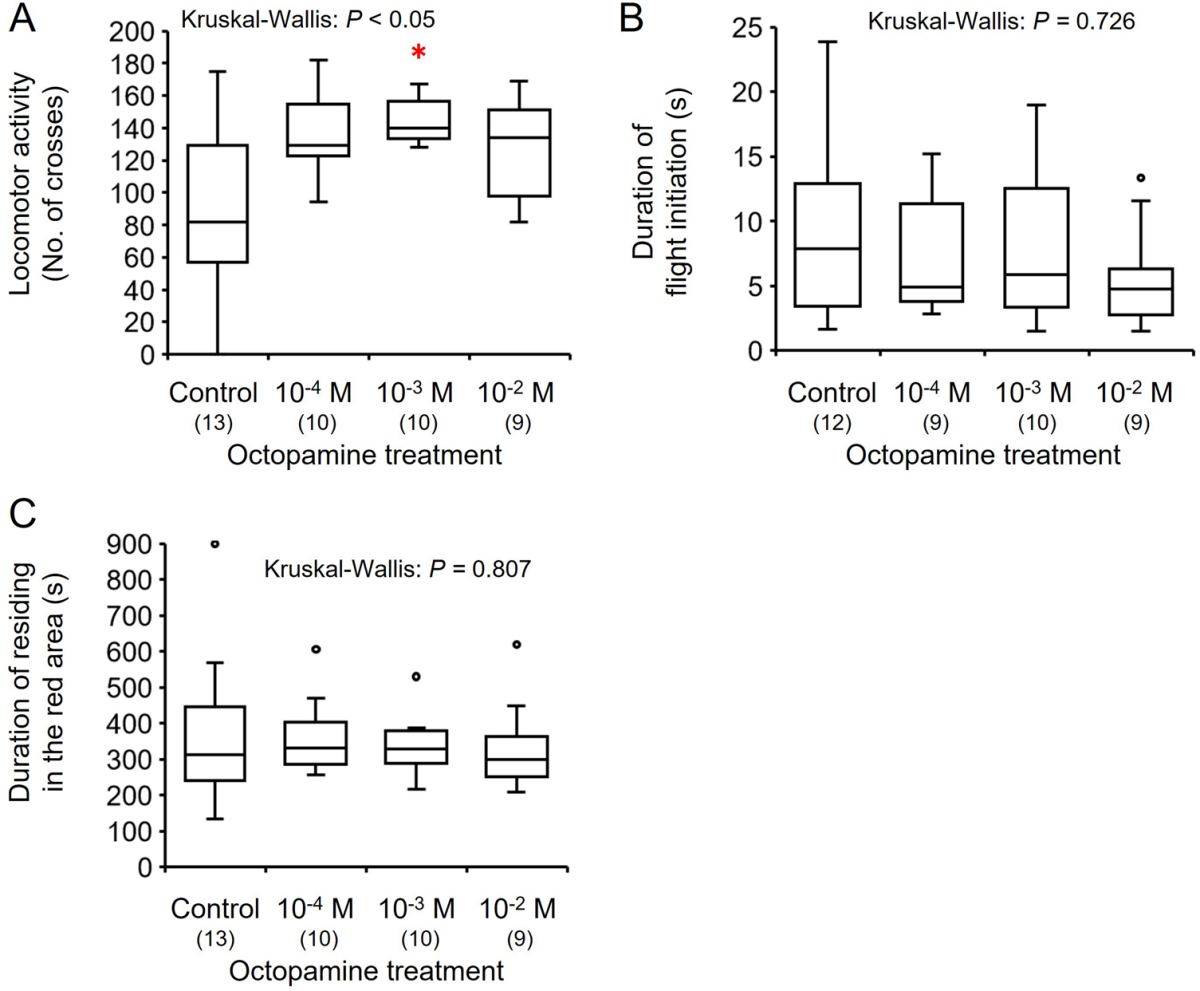
Effects of octopamine injection on behavioral activities of 4-day-old males in the bumble bee: (**A**) spontaneous locomotor activity, (**B**) duration of flight initiation, (**C**) duration of time residing in the red area as a light preference index. Locomotor activity, duration of initiation, and duration of residing in the red area are indicated by box plots. A significant difference according to the Steel test (*P* < 0.05) is indicated by an asterisk. Sample sizes are shown in parentheses.

The locomotor activity was significantly higher in individuals treated with 10^–3^ M octopamine than the control individuals (Kruskal–Wallis test: *H* = 8.764, *df* = 3, *P* < 0.05; Steel: *P* < 0.05, Fig. 5A). A similar trend was seen in males treated with 10^–4^ M octopamine, but significant differences from control males were not detected. The latency of flight initiation did not differ significantly between the control and octopamine-injected individuals (Kruskal–Wallis: *H* = 1.315, *df* = 3, *P* = 0.726, Fig. 5B). The duration of residence in the red area did not differ significantly between the control and octopamine-injected individuals (Kruskal–Wallis: *H* = 0.976, *df* = 3, *P* = 0.807, Fig. 5C).

### Effects of octopamine receptor antagonist (epinastine) on behavioral activity of bumble bee males

**Figure 6:**
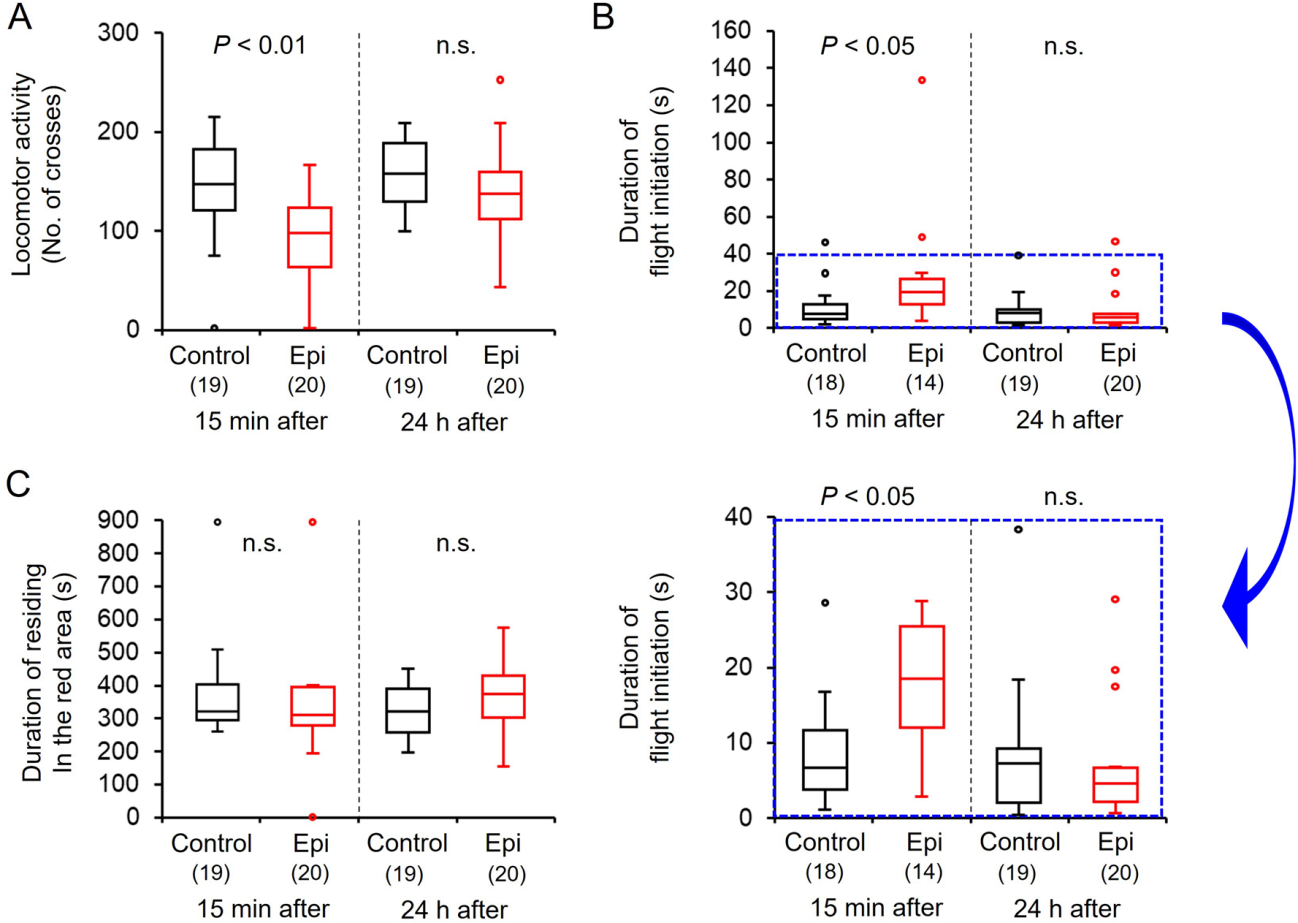
Effects of epinastine (octopamine receptor antagonist) injection on behavioral activities of 4-day-old males in the bumble bee: (**A**) spontaneous locomotor activity, (**B**) duration of flight initiation, (**C**) duration of time residing in the red area as a light preference index. Locomotor activity, duration of initiation, and duration of residing in the red area are indicated by box plots. Significant differences between the epinastine-treated and control groups were evaluated using the Mann–Whitney U-test. Durations of flight initiation are magnified in the boxes (blue dashed border). Numbers in bars indicate sample sizes.

The locomotor activity of males 15 min after epinastine injection was significantly lower than those of the control (Mann–Whitney U: *Z* = 3.304, *P* < 0.001, Fig. 6A). The activity of males did not differ between the control and treated males at 24 h after injection (Mann–Whitney U: *Z* = 1.447, *P* = 0.148, Fig. 6A), indicating the recovery from inhibition by epinastine. The latency of flight initiation in males at 15 min after epinastine injection was significantly longer compared with the control males (Mann–Whitney U: *Z* = 2.573, *P* < 0.05, Fig. 6B), and the latency in males recovered from inhibition at 24 h after injection and did not differ between the control and treated males (*Z* = 0.534, *P* = 0.593, Fig. 6B). The duration of residing in the red area did not differ significantly between the control and treated males (15 min: Mann–Whitney U: *Z* = 0.829, *P* = 0.407; 24 h: *Z* = 1.328, *P* = 0.184, Fig. 6C).

### Changes in levels of dopamine-related substances in the brains of honey bee drone pupae

**Figure 7:**
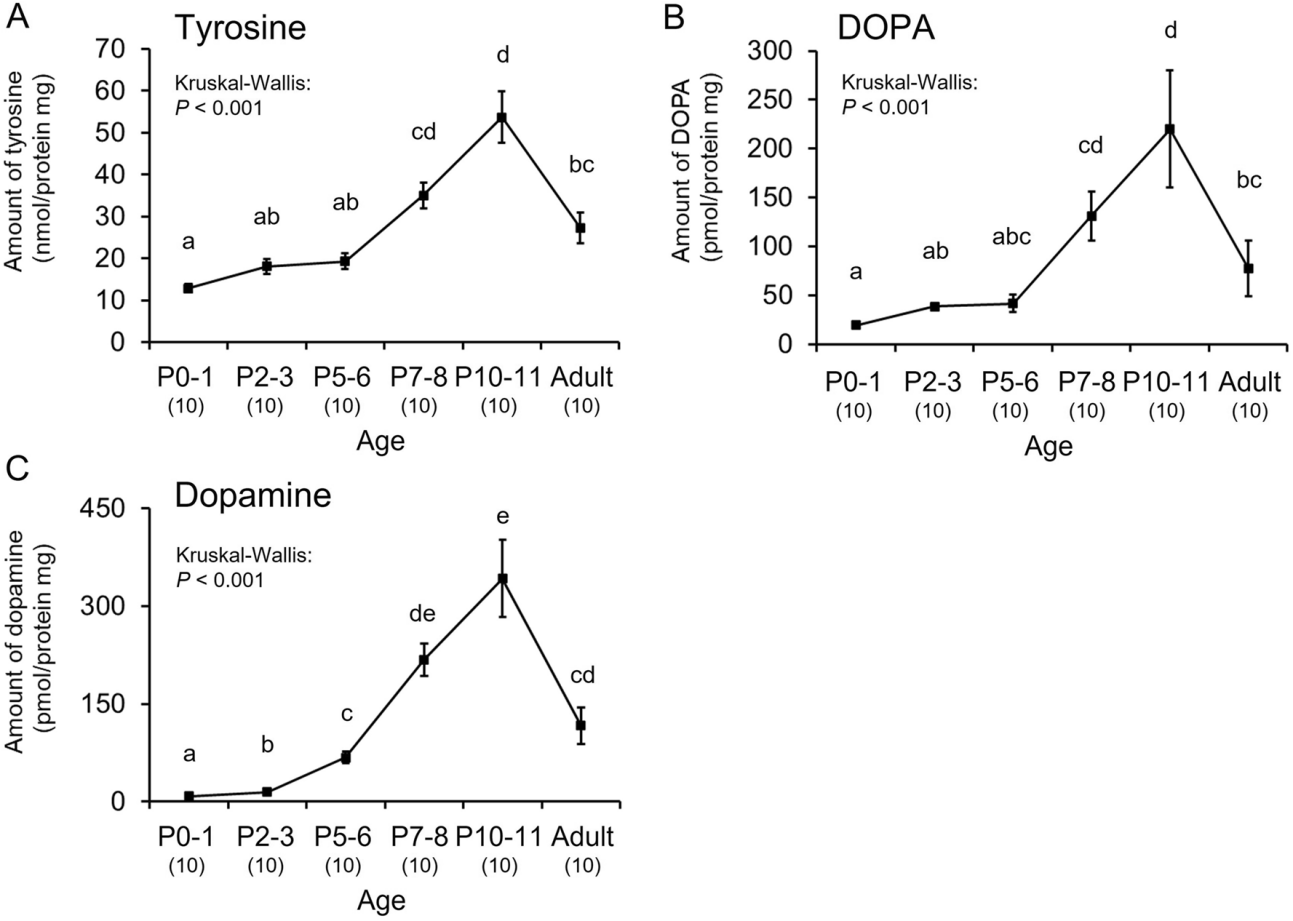
Age-dependent changes in dopamine-related substances normalized by brain protein in honey bee drones during the pupal stage. Changes in tyrosine levels (**A**), DOPA levels (**B**), and dopamine levels (**C**) are shown. Error bars indicate standard errors. Significant differences according to the Steel–Dwass test (*P* < 0.05) are indicated by different letters. Sample sizes are shown in parentheses below the age.

There have been no previous reports on the dopamine levels in the brains of honey bee drones during the pupal stage, so we determined the levels of dopamine-related substances in the brains of drone pupae. The changes in the levels of two precursors of dopamine (tyrosine and DOPA) and dopamine exhibited similar trends, and they depended on age during the pupal stage (Fig. 7A,B,C). The levels of these substances increased significantly with age and peaked just before emergence (P10–11), then decreasing upon adult emergence (tyrosine: Kruskal–Wallis: *H* = 38.542, *df* = 5, *P* < 0.001; Steel–Dwass: *P* < 0.05, Fig. 7A; DOPA: *H* = 35.346, *df* = 5, *P* < 0.001; Steel–Dwass: *P* < 0.05, Fig. 7B; dopamine: *H* = 51.459, *df* = 5, *P* < 0.001; Steel–Dwass: *P* < 0.05, Fig. 7C). A dopamine peak at around 8 days of age has been reported in adult drones in the honey bee^22^, so it appeared that the dopamine levels in the brain had two peaks from the pupal to adult stages.

## Discussion

Investigation of the physiological mechanisms that underlie the male reproductive behavior has been required for monophyletic clade species such as the corbiculate bees. In honey bee drones, the modulation of reproductive behavior by biogenic amines have been investigated^8,10,23^. However, there have been no previous reports of the biogenic amine levels in the brain and behavioral assays by application of biogenic amines in bumble bee males. The present study is the first determination of the functions of dopamine and octopamine in the behavioral activities of males in a primitive eusocial bee.

In bumble bee males, the octopamine level in the brain increased from the mid-pupal to adult stages. The locomotor and flight activities in adult males also increased with age up to sexually mature age (8 days old). These results suggest that octopamine is a potential neuroactive substance that coincides with age-related increases in behavioral activities. We then tested the effects of biogenic amines on the behavioral activities by drug injection. Octopamine injection increased the locomotor activity, whereas epinastine decreased the locomotor activity and increased the latency of flight initiation. Since epinastine acts through octopamine receptors as an antagonist, the results indicate that the blockade of octopaminergic signaling decreases the locomotor and flight activities, and therefore, octopamine enhances these activities in bumble bee males. In honey bee drones, the brain levels of octopamine and locomotor and flight activities increased with age^10,23^. Octopamine injection enhanced the flight activity in drones^23^. The changes in the octopamine levels and behavioral functions of octopamine have been shared between bumble bee males and honey bee drones.

In contrast to octopamine, the dopamine levels in the brain during the adult stage decreased immediately after adult emergence and dopamine injection did not affect the behavioral activities in bumble bee males. These results suggest that dopamine is not involved in the increase of the behavioral activities of bumble bee males at least at the day 4 of adult life.

**Figure 8:**
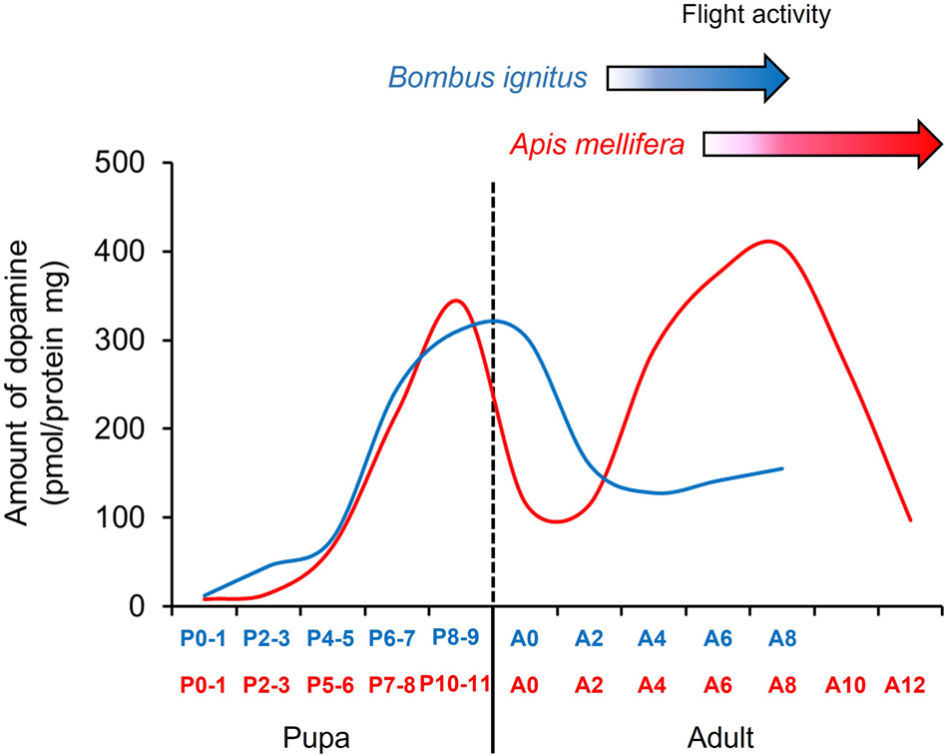
Comparison of changes in dopamine levels in the brains of honey bee and bumble bee males. Red and blue lines indicate dopamine levels in the honey bee (*Apis mellifera*) and bumble bee (*Bombus ignitus*), respectively. Dopamine data for the honey bee were obtained in the present study and studies by Harano et al.^22^ and Watanabe and Sasaki^32^.

We determined the changes in the dopamine levels in the brains of honey bee drones during the pupal stages to compare with those in bumble bee males. The dopamine levels in honey bee pupae increased with age and peaked immediately before adult emergence (P10–11). It was previously reported that the dopamine levels during the adult stage increased with age and peaked at 8 days after emergence^10,22^. Therefore, the dopamine levels in the brains of honey bee drones had two peaks from the pupal to adult stages (Fig. 8, red line). In contrast to the two peaks in the dopamine levels in honey bee drones, the dopamine levels in bumble bee males only peaked once from the mid-pupal to early adult stages (Fig. 8, blue line). This difference in the changes in the dopamine level during the adult stage between these two species may provide an important insight into the evolutionary process from a common ancestor to two species. In honey bee drones, dopamine enhances the locomotor and flight activities^10,23^, which correspond to a peak in the dopamine level at sexually mature age (A8). In solitary insects, dopamine enhances the locomotor activity and sexual behavior in *D. melanogaster*^29–31^, as well as the locomotor and flight activities in the large carpenter bee, *X. appendiculata*^24^. These results suggest that the dopamine effects on the locomotor and flight activities might be shared among males in several solitary species and the honey bee, but not in the bumble bee. It is required to be determined whether the ancestral characteristic is one or two dopamine peaks from the pupal to sexually mature adult stage.

Dopamine effects on locomotor and flight activities in bumble bee males may have been lost or unused with a lack of a dopamine peak during the adult stage. In honey bee drones, the dopamine peak at early adult stage is generated by effects of juvenile hormone (JH) and food intakes^8^. The JH titer in the hemolymph increases with age until sexual maturation as the dopamine levels in the brain^22,33,34^. JH enhances the gene expression of enzymes involved in dopamine biosynthesis and increases the levels of dopamine in the brain^22,23,32,35^. In bumble bee males, the JH titer may not increase during early adult stage and not stimulate dopamine biosynthesis. Intakes of foods containing tyrosine from nurse bees can also enhance the gene expression of enzymes of dopamine biosynthesis and increases the levels of dopamine during early adult stage in honey bee drones^32,36^. Adult males in the bumble bee do not receive foods from workers and may have less opportunity to intake tyrosine than honey bee drones. This may be another reason for the lack of an increase in dopamine.

## Conclusion

This study investigated the relationships between biogenic amines and the reproductive behavior of bumble bee (*B. ignitus*) males. We also compared the associated mechanisms in the bumble bee and honey bee to obtain insights into the evolution of the regulation of reproductive behavior. The octopamine level in the brain increased with age in adults in parallel with the locomotor and flight activities and light preference, whereas the dopamine level did not. Injection of octopamine and epinastine affected the locomotor and flight activities in opposite ways, but dopamine did not. These results suggest that the octopaminergic system enhances locomotor and flight activities involved in the reproductive behavior of bumble bee males. It has been reported that dopamine and octopamine promote the flight activity of honey bee drones. Therefore, the function of octopamine in promoting flight has been shared between bumble bee and honey bee males, but the behavioral function of dopamine with dopamine increase was not observed in the bumble bee males.

## Materials and methods

### Collection of pupae and adult of bumble bee and honey bee males

Commercially reared bumble bee (*B. ignitus*) colonies were kept in wooden boxes using the procedure described by Sasaki et al.^37,38^. Male pupae were collected from cocoons in 12 queenright colonies. To identify the pupal age, we defined a white male pupa with white compound eyes as a 0–1 day-old pupa (P0–1). We transferred 0–1 day-old pupae into plastic cups (Φ98 mm × 96 mm) kept at 28 °C under constant dark conditions, and then collected them at 0–1, 2–3, 4–5, 6–7, and 8–9 days after pupation. Newly emerged adult males (0 day-old males) were marked on the thorax with paint (Paint Marker, Mitsubishi, Japan) to identify their age. The marked males were returned to the mother colonies and collected from the colonies at a specific age. The collected males were used to measure the levels of biogenic amines in the brain and for behavioral experiments. The samples used for measuring the levels of biogenic amines were collected at 11:00–13:30. They were euthanized with liquid nitrogen and stored in liquid nitrogen until their analysis.

The pupae and newly emerged adults of honey bee (*A. mellifera*) drones were obtained from normal queenright colonies kept at an apiary in Tamagawa University, Tokyo, Japan. To obtain drone pupae, comb frames with equal areas of drone and worker comb bases were introduced into the colonies to allow workers to build comb cells on them. After the drone comb cells were capped, the frames were taken from the colonies and transferred into an incubator kept at 33 °C. The pupal age was identified based on the method described by Jay^39^. Newly emerged adults were also collected from the drone comb cells. Pupae and newly emerged adult drones were collected at 11:00–13:30, euthanized with liquid nitrogen, and stored in liquid nitrogen until dopamine-related substances were measured.

### Measurement of biogenic amine levels in the brain

Heads of the bumble bee and the honey bee stored in liquid nitrogen were dissected in 0.1 M phosphate buffer (pH 7.0) on a Peltier cooling unit (Kenis Ltd, Osaka, Japan) at approximately 4 °C under a microscope. The dissected brains were homogenized for 2 min with microglass homogenizers in 100 µL of ice-cold 0.1 M perchloric acid containing 0.1 ng/µL 3, 4-dihydroxybenzylamine. Each sample was then transferred into a 1.5 mL microcentrifuge tube and centrifuged at 15,000 × *g* and 4 °C for 30 min. Each supernatant was transferred into microvials for analysis by HPLC-ECD systems.

The HPLC-ECD system used for measuring the dopamine, octopamine, and tyramine levels developed by Sasaki et al.^40^ comprised a solvent delivery pump (PU-4180, Jasco, Tokyo, Japan), refrigerated automatic injector (AS-4050, Jasco), and C18 reversed-phase column (250 mm × 4.6 mm id, 5 µm average particle size; UG120, Osaka Soda, Osaka, Japan), and the temperature was maintained at 35 °C. The ECD (ECD-700, EICOM, Kyoto, Japan) was set at 0.85 V and employed at 35 °C. The mobile phase contained 0.18 M monochloroacetic acid and 40 μM 2Na-EDTA, which was adjusted to pH 3.6 with NaOH, and 1.62 mM sodium-1-octanesulfonate and 5% CH_3_CN were added to this solution. The flow rate was kept constant at 0.7 mL/min.

The HPLC-ECD system used for measuring the tyrosine and 3,4-dihydroxyphenylalanine (DOPA) levels developed by Matsuyama et al.^41^ comprised a solvent delivery pump (AS-4580, Jasco), refrigerated automatic injector (AS-4550, Jasco), and C18 reversed-phase column (250 mm × 4.6 mm id, 5 µm average particle size; MG, Osaka Soda), and the temperature was maintained at 35 °C. The ECD (ECD-700, EICOM) was set at 0.84 V and used at 35 °C. The mobile phase contained 83 mM citric acid monohydrate, 17 mM sodium acetate, 13 µM 2Na-EDTA, and 2.3 mM sodium-1-octanesulfonate, and 7% methanol was added to this solution. The flow rate was kept constant at 0.7 mL/min. In both HPLC-ECD systems, external standards were run before and after the sample runs to identify and quantify biogenic amines. The peaks were identified by comparing the retention time and hydrodynamic voltammograms with those obtained for the standards. Measurements based on the peak areas in the chromatograms were obtained by calculating the ratio of the peak area for a substance relative to the peak area for the standard.

To normalize the levels of the biogenic amines based on the protein contents in the brain, the amounts of protein were measured using the Bradford method^42^. The precipitated protein pellet obtained from the brain tissue after extracting biogenic amines was treated with the same procedures as described by Sasaki et al.^40^. The absorbance of each reacted sample was measured at a wavelength of 590 nm using a microplate reader (MPR-A100, AS ONE, Osaka, Japan).

### Locomotor activity, flight activity, and light preference measurements

Males collected from the mother colonies were individually transferred to a ring-shaped chamber (outer diameter 150 mm; inner diameter 90 mm) with a transparent sheet cover at 28 °C until the behavioral experiment (Fig. S4). The behavioral experiments were conducted as described by Sasaki et al.^43^. The transparent cover sheet was separated into four divisions by crosshairs and then covered by a red plastic sheet over the half of the transparent cover to provide a dark (red) area for insects in the chamber. After acclimatization for 5 min, the spontaneous locomotor activity of each bee was recorded using a digital video camera for 15 min. The number of crosses of the crosshairs was counted in the video data recording. In addition, not moving and walking individuals were counted. The duration of residing under the red plastic sheet was recorded for determination of light preference. After measuring the locomotor activity and light preferences, the latency of flight initiation was measured in a net cage (45 cm × 60 cm × 45 cm) at 28 °C, as described by Sasaki et al.^43^ (Fig. S4). The chamber containing a male was fully covered by red plastic sheet and transferred into the net cage. The red sheet was then removed to allow the male to fly spontaneously. The flight behavior during observation for 5 min after removing the red sheet was classified according to two levels as “not flying” (including wing flapping) and “flying.” The latency of flight initiation was recorded for the “flying” individuals.

### Effects of biogenic amine-related drug injection on behavior

Two µl of dopamine (Sigma-Aldrich) and octopamine (Sigma-Aldrich) solution (10^–4^, 10^–3^, and 10^–2^ M) were dissolved in 0.1 M phosphate buffer (pH 7.0) or 2 µl of 0.1 M phosphate buffer (control). Epinastine (TCI, Tokyo, Japan, octopamine receptor antagonist^44,45^) was dissolved in 0.1 M phosphate buffer to the concentration of 10^–2^ M. To examine the roles of biogenic amines in the locomotion, light preference, and flight behaviors of bumble bee males, prepared solutions were injected into the abdomens of 4 day-old males using a 10-µL microsyringe with a fine needle. The duration of acclimatization was 15 min after drug injection before observing locomotor activities.

### Statistics

All statistical analyses were performed using R software (ver. 4.1.2, https://cran.r-project.org/). Levels of biogenic amines were compared between groups using the Kruskal–Wallis test followed by the Steel–Dwass test for pairwise comparisons. Locomotor activities and durations of residence in the red area were also analyzed using the Kruskal–Wallis test followed by the Steel–Dwass test. Proportions of locomotor (“not moving” vs. “walking”) and flight (“not flying” vs. “flying”) activities were examined using Fisher’s exact test with Bonferroni correction. Behavioral activities of males were examined using the Kruskal–Wallis test followed by the Steel test (control vs. treatments). Behavioral activities of males treated with or without epinastine were compared using the Mann–Whitney U test.

## Supplementary Information

Supplementary Information 1.

Supplementary Information 2.

## Data Availability

All data used in the current study are available within the paper and its supplementary materials.
