# Supplementary material for: Behavioral roles of biogenic amines in bumble bee males
Source: Sci Rep. 2022 Dec 5;12:20946. doi: 10.1038/s41598-022-25656-7 (PMC9722695; doi:10.1038/s41598-022-25656-7)
Supplement: Supplementary file 2 — Supplementary Information 2. [file 41598_2022_25656_MOESM2_ESM.pdf]

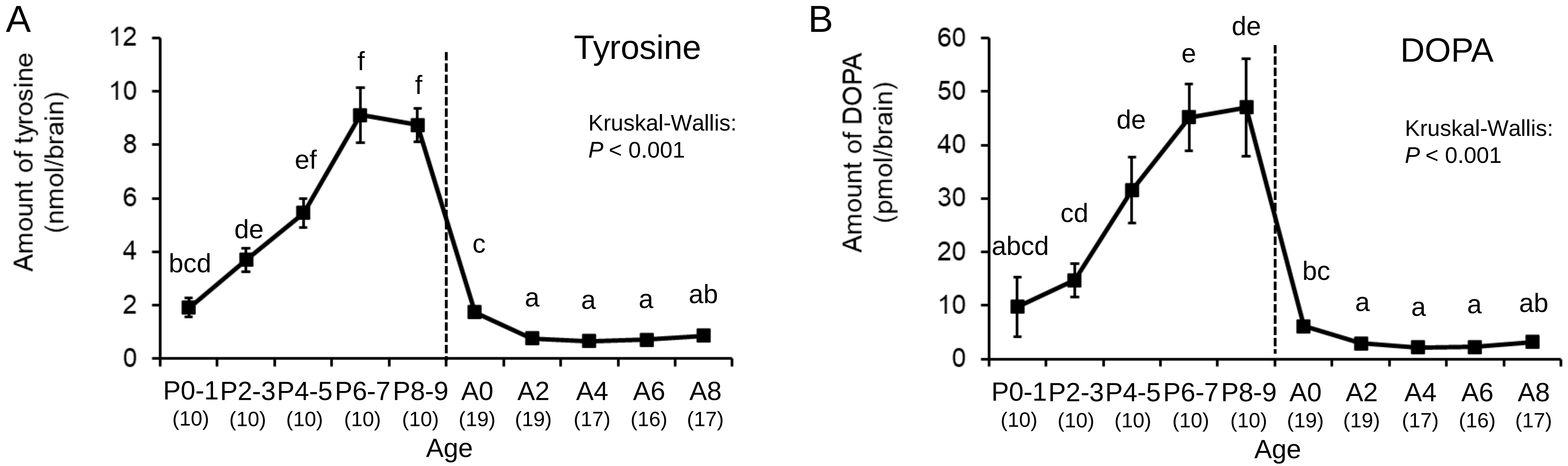

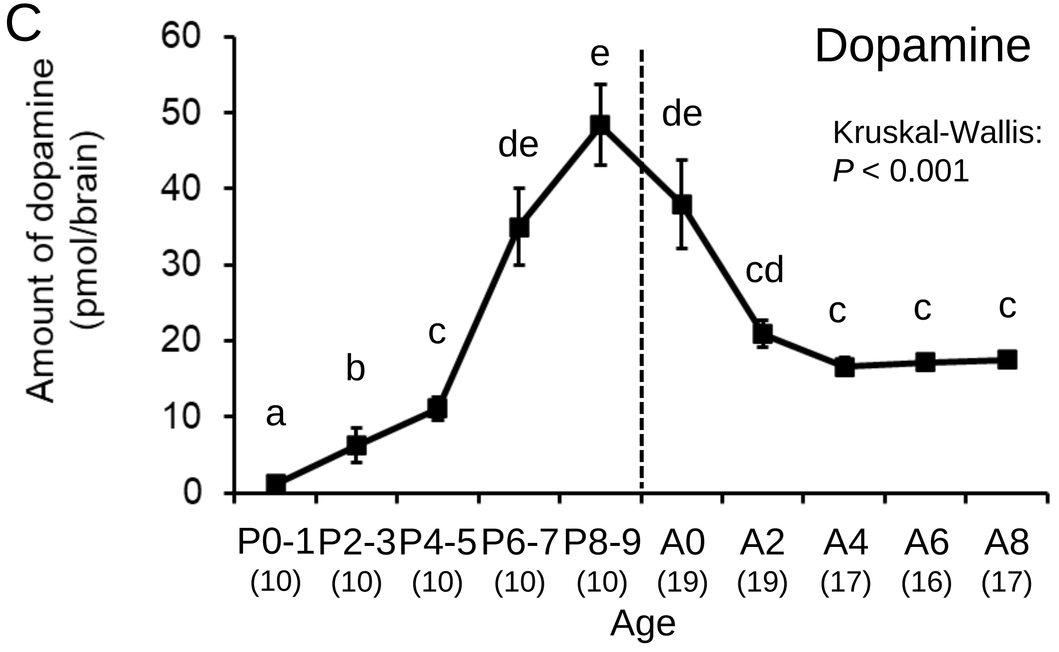

Fig. S1 Dynamics of dopamine substances in the brains in males of the bumble bee (per brain)

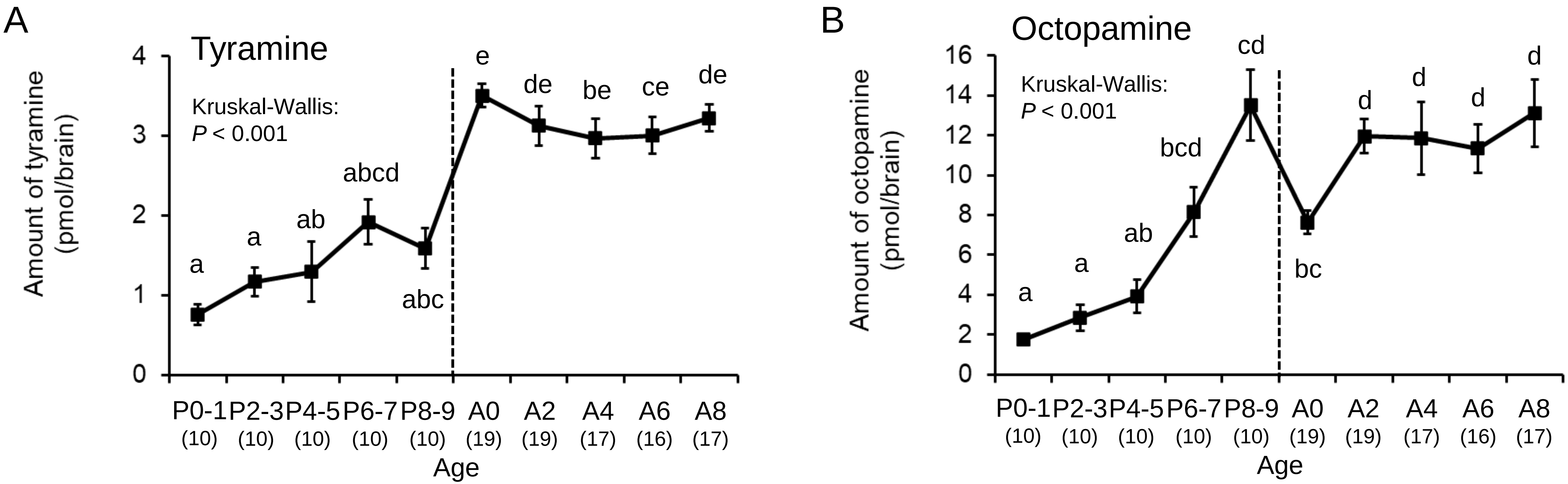

Fig. S2 Dynamics of phenolamines in the brains in males of the bumble bee (per brain)

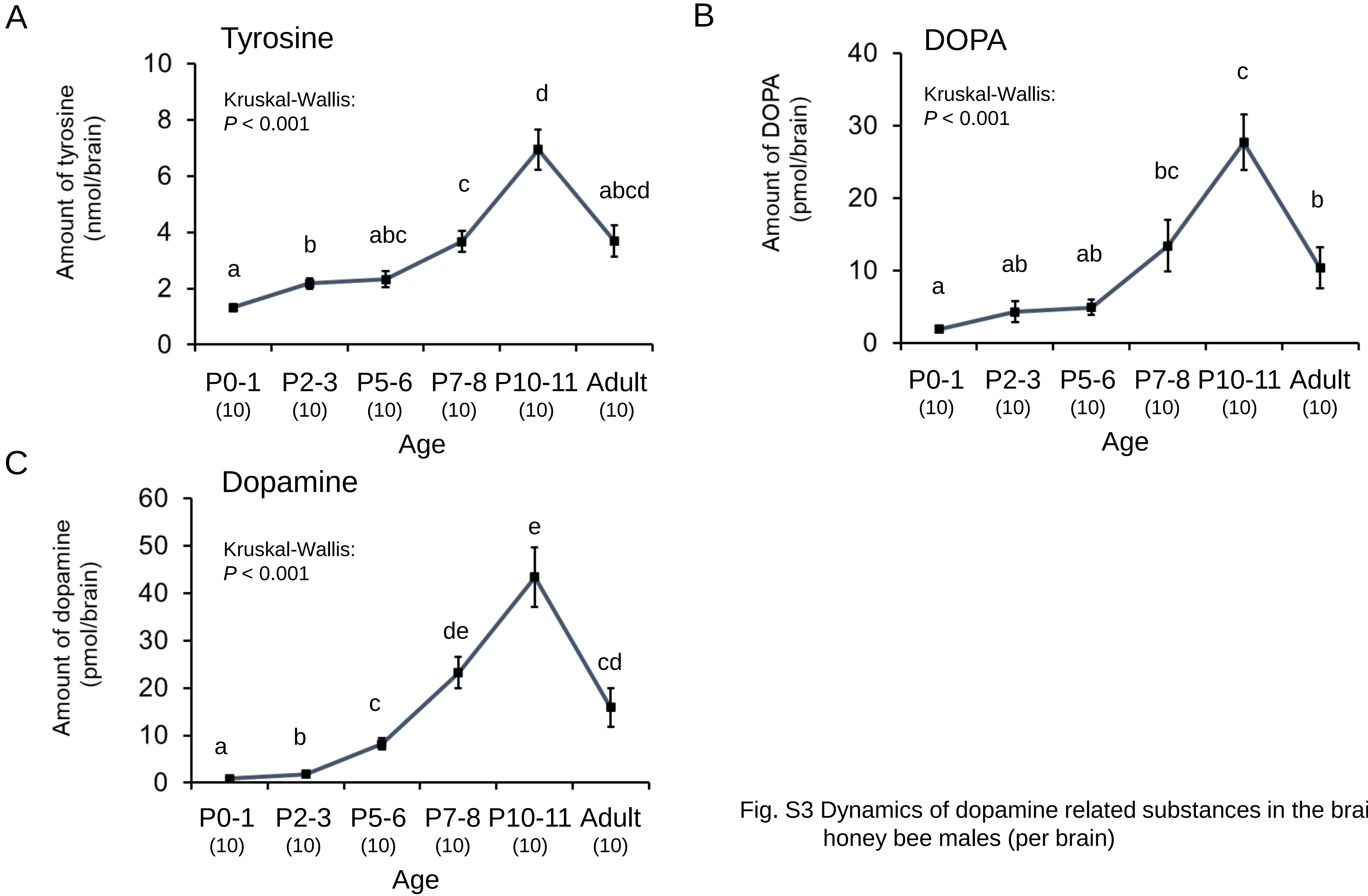

Fig. S3 Dynamics of dopamine related substances in the brains in honey bee males (per brain)

Behavioral experiments

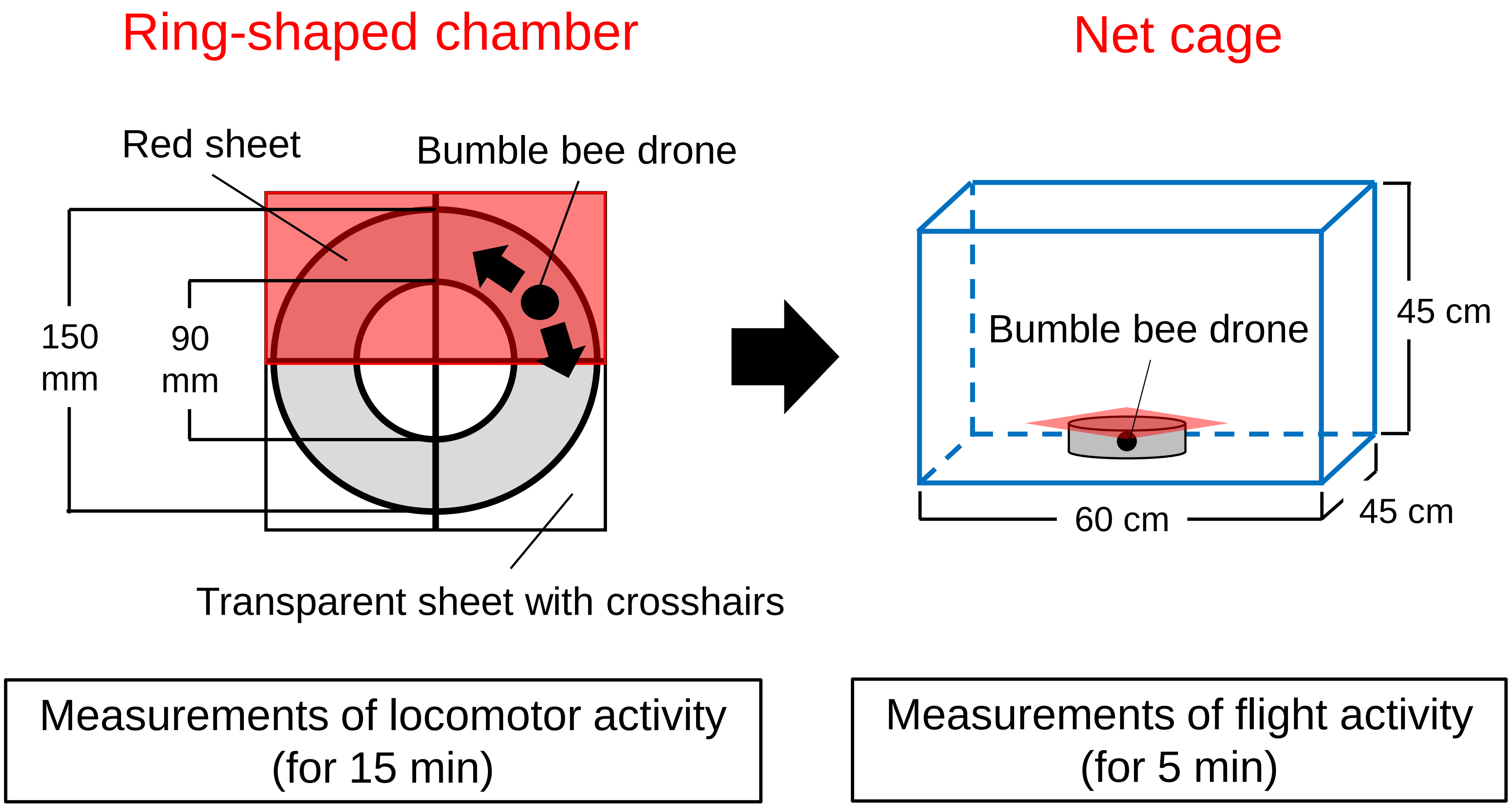

Fig. S4. The setup of the behavioral experiments. The locomotor activity was measured by the ring-shaped chamber for 15 min. Then, the chamber was transferred into the net cage to measure the fight activity for 5 min.
